# Occupational roles and risks of community-embedded peer educators providing HIV, hepatitis C and harm reduction services to persons who inject drugs in Nairobi, Kenya

**DOI:** 10.1371/journal.pone.0278210

**Published:** 2022-12-01

**Authors:** Linnet N. Masese, Natasha T. Ludwig-Barron, Loice Mbogo, Brandon L. Guthrie, Helgar Musyoki, David Bukusi, William Sinkele, Esther Gitau, Carey Farquhar, Aliza Monroe-Wise

**Affiliations:** 1 Department of Medicine, University of Washington, Seattle, Washington, United States of America; 2 Department of Epidemiology, University of Washington, Seattle, Washington, United States of America; 3 Department of Global Health, University of Washington, Seattle, Washington, United States of America; 4 Kenyatta National Hospital, Nairobi, Kenya; 5 Kenya’s Ministry of Health, Nairobi, Kenya; 6 Support for Addiction Prevention and Treatment in Africa, Nairobi, Kenya; The Technical University of Kenya, KENYA

## Abstract

**Background:**

In Kenya, harm reduction organizations have adopted evidence-based peer educator (PE) programs, where former people who inject drugs (PWID) serve as community health liaisons to engage PWID in HIV, HCV and harm reduction services. While PEs play an integral role in healthcare delivery, little data exists on their roles, risks and experiences working with PWID, which may be used to inform local harm reduction policy.

**Methods:**

PE’s from two harm reduction sites in Nairobi were randomly and purposively selected to participate in semi-structured in-depth interviews. Thematic analysis was conducted to characterize the expected versus actual roles that PEs employ while serving PWID, personal motivation and/or challenges and occupational health risks. Data was analyzed using Atlas.ti software.

**Results:**

Twenty PEs participated in the study. On average, PEs were 37 years of age, with 3 years of service. Female representation was 30%. Expected responsibilities included locating clients, establishing rapport, educating and escorting clients to addiction care facilities. Additional roles included attending to clients outside of work hours, escorting clients to medical appointments and facilitating patient-provider discussions. Occupational health risks included harassment by police and drug dealers, needle sticks, and close proximity to drug use environments that could prompt drug relapse. Despite these challenges and risks, PEs are motivated by their personal experiences of overcoming addiction with help from harm reduction programs.

**Conclusions/Recommendations:**

PEs play a vital role in HIV, HCV and harm reduction service delivery in Kenya, often exceeding their job descriptions by offering additional support to PWID. Recommendations include (1) advocating for PEs to provide patient navigation services, (2) promoting the use of post-exposure prophylaxis (PEP), (3) providing occupational mental health services to prevent drug relapse, and (4) collaborating with law enforcement to address harassment, with the ultimate goal of reducing HIV and HCV incidence among PWID.

## Introduction

Worldwide, people who inject drugs (PWID) account for 5–10% of individuals living with HIV [1]. PWID experience over 20 times greater risk of acquiring HIV than the general population due to practices such as sharing needles and other equipment used during drug injection [2]. PWID disproportionately contribute to Kenya’s HIV/AIDS and viral hepatitis epidemics. 7.5% of all new HIV infections in Kenya are attributed to PWID, who have an estimated prevalence of 14–20%, as compared with the general population prevalence of 4.9% [3]. The current size of the PWID population in Kenya is estimated to be around 20,000 individuals, the majority of whom are located in Nairobi, followed by Kenya’s coast [4]. Despite being at higher risk for HIV and viral hepatitis, PWID access and engage in services less frequently than other populations and this negatively impacts HIV and viral hepatitis prevention and treatment efforts [4, 5]. Globally, retention in care and adherence to treatment regimens are lower among PWID than among non-drug users [6–8] and sub-Saharan Africa has the lowest observed rate of antiretroviral use among HIV-positive PWID anywhere in the world [9]. Up to 30% of PWID in Kenya have never been tested for HIV and many who have tested positive are not engaged in care [3]. Consequently, targeted outreach and care efforts are urgently required to improve access to and ongoing engagement in harm reduction and other services among PWID [10, 11].

Innovative strategies are essential to identify, engage and treat key populations in an effort to reduce HIV and viral hepatitis infections and reduce onward transmission to sexual and injection partners. The World Health Organization (WHO) suggests adapting evidence-based harm reduction strategies and training former PWID to serve as community health liaisons and peer educators (PEs) to locate, engage and provide care to PWID communities [12]. Utilization of PEs has been well studied among people living with HIV/AIDS (PLHA) in Kenya and other settings with reported improvement in engagement and utilization of HIV care and ancillary services [13, 14]. Peer educators provide social and emotional support and can reduce stigma due to the connection from shared experiences [15, 16]. PEs also provide health and legal advocacy [17, 18], disseminate trusted information [19], create a sense of collective identity [20], facilitate engagement in research [18], and promote connection and communication with harm reduction and other health programs [21, 22]. Similar approaches have been adapted for PWID programs with encouraging results [23, 24]. Exposure to PEs results in increased access to harm reduction services and has been linked to higher reported levels of condom use and use of sterile needles among key populations [25, 26].

In response to the WHO recommendations, Kenya’s Ministry of Health (MoH) developed a comprehensive program [27] that details harm reduction services for PWID including needle and syringe programs (NSP), HIV testing, antiretroviral therapy, condom provision, hepatitis B and C testing, vaccination and treatment, and referrals to opioid substitution therapy (OST). The guidelines also recognize the benefit of using former PWID as outreach workers and PEs. PE programs are vital to establishing community rapport and gaining access to PWID, a vulnerable and often hidden community, in order to provide access to healthcare services [28]. PWID in Kenya currently access these services through a network of harm reduction programs operating in strategic areas of the country with the highest estimated PWID populations. These programs offer NSP, HIV testing, HIV treatment and care, condom distribution, peer education and counseling and social support services.

As PE programs for PWID gain recognition in Kenya and across the continent, their impact on high risk PWID behavior, and by extension, HIV and viral hepatitis infection is becoming more evident. However, little is known about their expected versus actual roles and responsibilities, the occupational health risks, and challenges encountered by PEs that serve PWID. Recent studies in Canada found that PEs experienced significant stigma, workplace inequities, and barriers to engagement with the drug use community [29, 30]; however, very few studies have evaluated PE experiences and challenges in low-resource settings. Through a secondary analysis of qualitative data collected for a parent study examining barriers to care among PWID, we aimed to characterize PE’s (1) expected versus actual roles and responsibilities in working to ensure PWID (clients) receive healthcare services, (2) professional motivations and challenges, (3) occupational health risks, and (4) recommendations for additional services and resources for PEs. Finally, we provide recommendations to assist healthcare policy makers and service providers leverage PEs to provide high quality harm reduction services to PWID, and to ensure that PEs are well-prepared and safe in the conduct of their work.

## Materials and methods

### Setting

Support for Addictions Prevention and Treatment in Africa (SAPTA) is a non-profit organization established in 2004 with a mission to provide substance abuse education, advocacy efforts, and training for harm reduction services and outpatient counseling and treatment in Nairobi, Kenya. SAPTA has four drop-in-centers where PWID can access social services (e.g., food, shower and laundry facilities), HIV and HCV testing and referrals for HIV, HCV, OST care and ancillary health services (e.g., wound care). SAPTA locations are in areas that have a high prevalence of injection drug use; however, to prevent contributing to the marginalization of these communities, location names will remain anonymous.

SAPTA’s PE program incorporates the Kenya MoH PE Training Manual that uses evidence-based harm reduction principles to train former PWID to deliver health education messaging and harm reduction services to hidden PWID communities [31]. The 5-day pragmatic training curriculum includes: basics of drugs and drug harms, harm reduction services (NSP, OST), behavior change communication, outreach and relationship skill-building, and identification and mobilization of PWID. The course is followed by a shadowing component, where newly trained PEs assist senior PEs to provide harm reduction services in the community. While PEs are employed by SAPTA, this employment is informal and contractual, and PEs are compensated with a stipend rather than salary with benefits.

This study was a collaboration between researchers, SAPTA leadership, government program managers, and HIV/HCV clinicians that were involved with the conception of research questions, interview guide development and the dissemination of findings. The *a priori* parent study aims included understanding HIV/HCV barriers and facilitators to care, and resource recommendations to improve service uptake, through the lens of community-embedded PEs. During early team discussions, we identified an emergent theme surrounding the expected vs actual occupational roles that PEs performed, occupational risks PEs encountered and resource recommendations to improve the working conditions of PEs. This informed a secondary data analysis, led by the lead author (LNM), which focused on the occupational roles, motivations and risks PEs encounter while delivering harm reduction services.

### Sampling & recruitment

Through SAPTA, we established a sampling frame of 60 PEs working at two drop-in centers in September 2017. Study eligibility included being 18 years or older, employed by SAPTA as a PE at the time of recruitment, English or Swahili-speaking, and being willing and able to provide informed consent. Random and purposive sampling techniques were applied, where we randomly selected PEs from a SAPTA roster and oversampled female PEs, which was essential to the parent study aims.

Twenty participants completed in-depth interviews from November through December 2017. A standard script was used to explain the study purpose and procedures, with all participants providing written informed consent in Swahili or English. Study protocols ensured that PEs disclosing active engagement in substance use were connected with an addiction counselor and appropriate follow-up ensued. To our knowledge PEs were not actively engaged in substance use at the time of their interviews. Upon interview completion, PEs were reimbursed KES 400 ($4 USD) for their time and transportation. All study procedures and materials were approved by the University of Washington Institutional Review Board (Seattle, WA, USA) and the Kenyatta National Hospital Ethical Review Committee (Nairobi, Kenya).

### Data collection & management

Through a collaborative process, a semi-structured, in-depth interview guide was developed by several co-authors, including SAPTA leadership (S1 Appendix). The interview guide aimed to elicit information on PEs personal and professional experiences surrounding (1) PWID access and utilization of HIV, HCV and addiction care services; (2) treatment by law enforcement and medical providers; and (3) suggested resources for providing harm reduction services. The interview guide, was translated into Swahili by the study coordinator (LM), who is multilingual and bicultural, with several years of experience working with PWID. Prior to data collection, the interview guide was back-translated by a SATPA manager (EG) and pilot tested with two PEs to ensure colloquial terms were incorporated appropriately.

One bilingual (English/Swahili), Kenyan, female interviewer with masters-level training and extensive experience conducting qualitative studies, received training on study protocols and conducted all interviews. All interviews were audio recorded and lasted between 25 to 45 minutes, which were translated and transcribed verbatim. The interviewer took detailed field notes to capture the physical surroundings, body language and emotional condition of each participant. Study team members met on a weekly basis to refine the interview guide, to discuss emergent topics (i.e., reciprocation) [32], and to initiate preliminary analysis discussions. Data collection ceased after conceptual saturation had been reached and additional interviews would not elicit new information [33], which was determined to have occurred after 20 interviews. ATLAS.ti, Version 8, was used to analyze all transcripts, field notes and memos into one integrated system.

### Data analysis

This study borrows from the thematic analysis methods described by Guest et al. [34]. Through ongoing team discussions, we found several emergent themes discussed frequently by participants, which prompted the first author (LNM) to further explore these topics via a separate secondary thematic analysis (S1 Fig). Emergent themes included PEs’ (1) expected versus actual responsibilities, including strategies they use to ensure their clients receive healthcare services, (2) professional motivations and challenges, (3) occupational health risks and (4) recommendations for additional services and resources for PEs. Transcripts were open coded by the lead author (LNM), with every-third transcript reviewed by researcher team members (NLB, AMW, LM) to ensure codes were applied consistently. Weekly team discussions included emergent themes related to study aims, codebook development, and code definition refinement as an iterative process. Recurring concepts, were organized into typologies and classification schemes, and upon further analysis, merged into themes. Isolated coding concerns were resolved through team member discussion and further refinement of coding parameters. To ensure reliability and validity in our qualitative analysis, we relied on triangulation wherein we probed for deeper understanding of the issues surrounding PE occupational risks among PWID using multiple methods including open coding for transcripts, which included multiple in the reviewing and interpretation of the data, and reviewing interviewer field notes to develop a more valid and reliable construction of reality [35]. Results were discussed with SAPTA leadership and government program managers on a regular basis, to ensure findings could be applied to the ongoing development of PE training programs. The following illustrative quotes were selected based on their ability to clearly demonstrate themes. Culturally relevant pseudonyms were assigned to protect respondent confidentiality, while humanizing our rich content through quotation excerpts [36].

## Results

We enrolled 20 PEs; 6 (30%) were female (Table 1). The participants’ mean age was 37 years. The average duration of serving as a SAPTA PE was 3 years. More than half (65%) of participants were married. Several reported having some college (30%) or some secondary education (30%).

**Table 1:** Characteristics of study participants.

Characteristic	In-depth Interviews (N = 20)
	n (%)
Sex	
Female	6 (30%)
Male	14 (70%)
Age (Mean)	37 (SD* = 6.6)
Level of Education	
Completed College	1 (5%)
Completed Primary	3 (15%)
Completed Secondary	4 (20%)
Some College	6 (30%)
Some Secondary	6 (30%)
Married	
Yes	13 (65%)
No	7 (35%)
Average years of service (range)	3 years (2 months– 6 years)

*SD–standard deviation.

### Roles and responsibilities of PEs serving PWID

Participants received extensive training on their expected roles and duties prior to employment (Table 2). PEs described in detail their obligations as specified in their job description. In the course of reviewing the interview transcripts, we also discovered responsibilities that PEs were performing in addition to their specified duties.

**Table 2:** Summary of occupational roles and risks of PE work in Kenya.

EXPECTED ROLES	ACTUAL ROLES	HAZARDS/RISKS
Locating clients (i.e., PWID)	Addressing client calls after hours	Harassment by police, drug dealers and PWID
Establishing community rapport	Escorting clients to medical appointments	Unintentional needlestick injuries
Harm reduction services (i.e., distributing SEP)	Facilitating discussion w/ providers on behalf of clients	Exposure to triggers, mental health stressors and potential relapse
Escorting clients to SAPTA for HIV, HCV and addiction services	Personal income used for transport to medical facilities	

#### Expected roles and responsibilities

Participants were clear about their usual roles and responsibilities. They described their duties as being locating clients; establishing community rapport; providing harm reduction services; and educating and escorting clients to SAPTA for HIV, HCV and harm reduction services. Each PE was assigned a number of clients (20–40) who they visited on average 2–4 days in a week. During their time with clients, PE’s emphasized: (1) primary prevention education on safe injection practices to prevent HIV and viral hepatitis transmission; (2) secondary prevention, by encouraging clients to test for HIV and HCV, and receive additional social services available at SAPTA and (3) assistance with tertiary prevention services, by providing medical appointment coordination. They distributed clean injection equipment (syringes, cotton swabs, metal containers, water packets and alcohol swabs) and condoms to their clients at the various drug dens in the city, which helped them build trust and rapport in the community. In addition, PEs provided documentation for each visit, including number of NSP kits and condoms they distributed at the visit.

“*What we do every week*, *everyone as a peer educator has a group of clients that we follow*, *around 40 of them*. *You are supposed to ensure that your clients have received the services that you have been assigned to deliver*. *Per week we would pick these condoms and syringes and take [these] to them*. *You ensure that they have condoms and needles and after every one month we bring them to the center to be tested*. *We also do follow-ups after three months*.*”* Kariuki, 47, M, 3 years of service

#### Additional roles and responsibilities

Peer educators often reported incidents where they performed tasks that were outside the scope of their training and duty in order to provide exceptional care to PWID. If their clients were unwell, they visited them at home and also called SAPTA leaders to arrange for transport to the drop-in-center or to a hospital. At the hospitals, they ensured that participants had been attended to, facilitated discussions with providers on behalf of clients and arranged for financial costs to be waived. In some cases, this involved fielding calls after hours, visiting hospitalized clients on a daily basis until they were discharged home, using personal funds to transport clients to hospital appointments, buying medications prescribed by doctors, and providing food when clients were hungry, fearing that providing money would trigger drug use. Kizito described the urgency and effort it took to assist clients during medical appointments, which he felt obligated to do:

“*Now if the client was my client*, *I have to take him to the hospital and take care for them until the day they are discharged*. *You have to visit*, *sometime stay along with them [overnight] and may be in the morning I can come to the drop-in-center and then go home*, *change and then go back*. *It is very hard sometimes*.*”* Kizito, 44, M, 2 years of service

Similarly, Wairimu described having to assist clients who fell victim to violence and the need to act swiftly, regardless of her personal penalty to fund the client’s medical needs. She described the need to assist her client and transportation barriers:

“*So*, *at night it is hard because they don’t even know where we live*, *sometimes they go stealing and they are beaten so when we come in the morning to SAPTA that is when we are informed*. *Sometimes transport is a challenge because I use my own money and maybe that day I don’t have enough*. *For now*, *we don’t have transport to take them to the hospital*.*”* Wairimu, 27, F, 3 years of service

Peer educators in this study surpassed expectations and defined job descriptions to serve PWID. It is evident that they sympathize with PWID and are motivated to support and advocate for them. They use their knowledge and experience of the community and health care systems to help PWID navigate these complicated systems. This may reflect their understanding of the plight of PWID having lived through similar experiences.

#### Occupational risks for peer educators

PEs identified several risks that they encounter in their daily work, namely: police harassment, harassment by drug dealers, needlestick and sharps injuries, being robbed and the potential risk of relapsing back into drug use.

### Police harassment

Six participants identified police harassment as an occupational risk of working as a PE serving PWID, especially when visiting the drug dens (specific locations where drugs are bought, sold and consumed). When police raided the dens, PEs were mistaken for drug dealers and were rounded up and taken into police custody along with the drug dealers. It was only after the SAPTA officials called the police department that they were released. Such experiences caused undue stress to PEs as described by Martin, a relatively new PE.

“*Challenges like going to the drug dens where they are selling*, *and maybe policemen had come to raid the dens*. *You can explain [to the policemen] but sometimes they can still arrest you*.*”* Martin, 50, M, 1 year of service

Not all PEs reported experiencing police harassment, and some felt that the police harassment was on the decline as a result of SAPTA’s efforts to hold frequent conversations with the police department to educate them on the services that PEs provide. Participants also reported having work identification cards that they carried and produced during interactions with police to prevent police from harassing them.

“*When we started this program*, *we could get some of us being caught [arrested] by police when we are at the dens*. *But SAPTA talked to the police department*, *so this time is not so bad like before*.*”* Mogaka, 47 M, 6 years of service

### Harassment by drug dealers

In three cases, participants reported being harassed by drug dealers who were frustrated with PEs for providing harm reduction services to their clients, hence interfering with their potential source of income. PWID who visited SAPTA received addiction counselling, and if they were enrolled in the methadone program, they were likely to stop using drugs. This meant less money for the drug dealers and resulted in some hostility towards PEs.

“*Sometimes you have to hide so that you can talk with people at the den*, *or when distributing the kits sometimes we hide because the drug dealers see us as a hindrance to their business*. *They will ask you why you get people [PWID] and put them in methadone program*, *and they leave drugs*. *They say we make their business go down when people go to Ngara [methadone clinic]…*.. *There was a time some peer educators from here [drop-in-center] went there [drug den] and they were beaten*.*”* Moha, 34, M, 2 years of service

### Needlestick and sharps injuries

Another occupational hazard is the possibility of needlesticks and sharps injuries. Two PEs described the dens as being littered with used needles, syringes and injecting equipment. Some described how some PWID after injecting drugs find it hard to get up and safely dispose of used needles because they are intoxicated. Moha described the risk of inadvertently sitting on or being pricked by a used needle at the drug den with the possibility of contracting infections:

“*Sometimes you go to give people advice [at the drug den] and you sit on a needle which has been used*, *you can pick disease from there because needles are all over [the drug dens]*. *All those are risks that should be looked into*.*”* Moha, 34, M, 2 years of service

### Experiencing robbery

Three PEs described the possibility of being mugged by PWID for money or items to sell for drug money.

“*… if you have your phone and receive it there*, *you will be surrounded by almost 4–5 people and they take away your phone and they go and sell it for stuff [drugs]*. *Sometimes when you get there*, *they think you have money; they can strangle you……*. *you know it has been hectic*.*”* Moha, 34, M, 2 years of service

### Drug use relapse

There were both direct and indirect descriptions of occupational mental health challenges, which could prompt drug use relapse. Three PEs directly described the risk of temptation to use drugs again as a result of interacting with others using drugs, while other discussions included having to overcome a traumatic experience related to a client death or relapse. Having recovered from drug use, these were noted to be environments where the temptation to use drugs was rife, with a risk of relapsing into drug use while working with PWID.

“*What I can say if someone wants to change*, *they must change the people who they are hanging around with because you cannot stay with the same people and you want to recover*, *they will take you back*. *If you take methadone and you go back to the same place it will not help*.*”* Hawi, 34, F, 3 years of service

“*…but if you have not decided*, *even when you take methadone*, *you just get back to using heroin*. *You see your friends using heroin and you get the urge to use it too*.*”* Tito, 40, M, 3 years of service

These quotes highlight the precarious conditions PEs serving PWID work in, and the need for interventions to address these occupational hazards as a means of protecting PEs and ensuring that these risks are not a barrier to building strong sustainable PE teams. For example, training police officers on knowledge related to drugs and harm reduction services (clean needles and opioid substitution) may improve their knowledge and attitudes towards PWID leading to fewer arrests and improved working conditions for PEs.

### Motivations and challenges experienced by PEs

Nearly all PEs (n = 17) were motivated by the desire to help others, just as they had been helped (Table 3). PEs in this study reported that they were initially participants in the harm reduction program, then began using methadone and eventually as they recovered from drug use they felt compelled to help other PWIDs work toward recovery. Participants noted that the guaranteed stipend at the end of the month was helpful in paying for housing, buying food and in some cases saving money for the future. However, they also reported financial and emotional challenges associated with work as a PE.

**Table 3:** Summary of motivations, challenges and recommendations for additional services and resources for PE.

MOTIVATIONS	CHALLENGES	RECOMMENDATIONS
The desire to help others	Stipend provided is inadequate necessitating additional work to supplement income	Additional training to enhance their work as PE
Stipend provided at the end of the month	Emotional burden of watching PWID relapse or die	Access to reliable transportation to easily and efficiently serve PE
		Distinctive identification cards for PE
		Risk allowance

#### Financial challenges

Although PEs desire to serve PWID and to continue in this line of work, twelve PEs reported holding side jobs to supplement the income they received as a PE. They noted that although the amount received as stipend was good, it was not enough to meet their monthly expenses. The request for a stipend raise was echoed by several participants in this study. Some of the side jobs that PEs reported were selling vegetables, selling second-hand clothes at the market, waste collection, and working as a mountain guide.

“*As a peer educator what I would request is that these people should think about us*. *This is on the side of the money*, *if they can add us some money that would be great*. *You see with this kind of money you have to look for something else on the side to do*.*”* Mwangi, 43, M, 3 years of service

“*Most Saturdays I go with the waste collecting vehicle and I get my KES 500 that day and pick plastics waste*.*”* Onyango, 32, M, 2 years of service

When asked if they would leave work as PEs for other jobs if opportunities arose, 11 PEs said they would not leave this work as they enjoyed working with PWID. They felt that they understood PWID well given past experiences as PWID and were willing to help PWID receive harm reduction services. For some, working as a PE was noted to be a calling, and although they knew they could undoubtedly obtain employment in other fields or places, they were dedicated to helping others just as they were helped. Rita described her motivation for continuing to work with clients, despite the occupational health risks she encounters:

“*You know*, *it is not that you cannot get other places but because SAPTA has made me to be what I am today and I would want to help someone else and that person also get to where I am*.*”* Rita, 38, F, 3 years of service

Inadequate compensation, minimal or nonexistent job benefits and inadequate budgetary allocation for PE activities could undermine PE recruitment, retention and harm reduction program efforts to reach PWID. Addressing remuneration, benefits and setting aside funds for outreach activities will ensure harm reduction organizations can build and sustain strong PE networks.

#### Emotional challenges

Nearly all peer educators (n = 17) reported the emotional burden of working as a PE. Watching their clients die from drug overdoses or infections was reported by some PEs as emotionally draining. Some clients finally succumbed to infections due to multiple interruptions to treatment. Others reported clients who were beaten to death for stealing and unfortunately, PEs were unable to help them. Kizito described the mental health strain that accompanies his role:

“*It is very painful that sometimes*, *I have lost some of them…*. *Yes*, *they succumb and pass away it is very distressing*.*”* Kizito, 44, M, 2 years of service

Onyango described a client escaping from the hospital multiple times to look for drugs while undergoing treatment because of withdrawal symptoms and PEs having to bring them back to the hospital numerous times. While assistance to clinical appointments is part of a PE’s job responsibilities, there is an unsaid mental health burden that accumulates when clients perish:

“*…*. *there is a guy there who died of hepatitis and he had a big stomach and every time he was taken to the hospital to get the water [ascitic fluid] out*, *he would escape from the hospital*. *He would stay there for two days and he will escape to come and just get high and he smokes then he realizes that he has done something stupid*. *Then we would call the ambulance and it will come and take him back*. *He got too sick and unfortunately he died*.*”* Onyango, 32, M, 3 years of service

Working with PWID carries significant emotional anguish for PEs. They invest time, energy and resources to work with PWID with the hope that they will overcome their addictions. Losing clients they have worked with and built relationships with can have long-term psychological consequences.

#### Recommendations for additional services and resources for PE

The final theme was on recommendations by the PEs for additional services and resources to enhance their work experience as PEs and to better equip them to serve PWID. Peer educators suggested basic life and emergency skills training, access to an emergency vehicles or ambulances, high visibility jackets for easy identification and a risk allowance given the risks they face in the line of duty.

### Additional training

When asked what additional services or resources might enhance their work, three PEs mentioned that receiving training on basic life support and first aid would be useful. They felt that this would prepare them to deal with emergency situations such as overdoses and other medical emergencies while in the field.

“*Yes*, *for the peer educators if they can be trained as nurses*, *it can be very helpful in areas like First Aid*. *This is very interesting because we can take care of them in the field…*. *Yes[if] we have the skills and the kit we can do it when needed*.*”* Kizito, 44, M, 2 years of service

#### Access to transportation or transportation reimbursement

Five PEs suggested that the availability of a car purchased through SAPTA to facilitate transportation of PWID from homes or dens to a hospital and for urgent supply of NSP kits. In addition, for those that have to use personal finances to fund transportation, they should be promptly reimbursed. Having backpacks was also recommended for easier transportation of needles, syringes and condoms.

“*There are many things we can do*, *for example having an emergency car*, *also most junkies sleep in an open place and sometimes they might be in need and we have to call MSF [Medicins Sans Frontiers] to get an ambulance and they might tell you to wait and during that time the person is in distress*. *If an emergency car is available*, *it will make things move faster to take him/her to the hospital*.*”* Kariuki, 47, M, 3 years of service

#### Distinctive identification

Five PEs suggested providing distinctive identification, including employment cards, vests, and/or jackets. Mogaka’s suggestion was to give PEs high visibility jackets that would serve as an identifier to prevent them from being rounded up by police when they raided drug dens.

“*But there is another challenge because you can be speaking to someone and that person is coming from committing a crime*. *By the time you convince the police that you are helping this person*, *you might find yourself locked up as well*. *So*, *we would request that when someone is going to the den*, *if we can be given those jackets (like the ones with reflectors) just to identify who you are*.*”* Mogaka, 47 M, 6 years of service

#### Risk allowance

Fifteen participants requested salary increases, but one additionally suggested a risk allowance given the occupational risks of this job such as police and drug dealer harassment, and the risk of needle pricks or provision of security to prevent drug dealers from pursuing them.

“*So*, *it will be nice if we get risk allowance because it is a place you can also be easily killed*.*”* Moha, 34, M, 2 years of service

Not only are PEs willing to exceed expectations, they also provide actionable suggestions to improve efficiency of their outreach activities. For example, they recognize their knowledge gaps and are willing to receive additional training to better serve PWID. As mentioned earlier, adequate budgetary allocation for remuneration and outreach activities will ensure uninterrupted harm reduction services for PWID.

## Discussion

This study among PEs who were former PWID provides valuable insights into the roles, responsibilities and risks associated with outreach and PE work among PWID. Overall, PEs reported their main duties as being: locating clients, educating them on harm reduction, providing HIV/HCV education and prevention, distributing safe injection supply kits, and escorting clients to the drop-in-center for additional counseling and testing. In several instances, PEs noted that they provide additional services above their scope of work and training, including navigating healthcare systems, servicing calls after hours and using their own money to help clients. This led to calls for longer working hours at the drop-in-centers, ambulance availability, and PEs requesting to be trained in first aid so as to respond to emergencies in the field.

Peer educators have been leveraged with great success among essential populations such as PLHA to promote access to HIV care [13, 37]. Use of PEs to access difficult-to-reach communities such as PWID is gaining acceptance; however, few studies have focused on PEs among PWID and more so, the roles and risks of working as a PE among PWID [17]. Peer educators who serve PWID play a vital role in HIV, HCV and harm reduction service delivery, through lived experiences and intimate knowledge of community needs [28]. Findings from our study indicate that PEs were willing to exceed their job expectations to serve PWID as they recognized and intimately understood the challenges facing PWID communities, having lived that experience. Studies have shown that these connections and shared experiences can lead to reduced stigma and may ultimately lead to behavior change such as using sterile injecting equipment and using condoms [25, 26]. Peer educators leverage their trust, community knowledge and experience to mitigate difficult clinical environments resulting in improved outcomes for their clients including reduced stigma, discrimination, and criminalization; increased access to services; safer injecting practices; and decreases in HIV prevalence [17, 38, 39]. Utilization of PEs is however one of many strategies to reach out to PWID. Countries within Africa and globally have successfully applied community-based approaches to provide a variety of health services to PWID including, HIV and viral hepatitis testing, supervised injection facilities, needle exchange programs, and OST [40, 41]. However, PE programs hold several advantages over community-based programs, including reduction of stigma and promotion of effective communication.

Peer educators in this study were enthusiastic about their roles and suggested additional training to expand the scope of their duties and prepare them for situations they occasionally face. We noted that PEs were navigating the health care systems and providing care in the field without significant training on these roles. Expanding PE programs by providing evidence-based training to PEs in navigating healthcare systems and responding to emergencies in the field would allow PEs to confidently and safely assume the role of peer navigators. Indeed, WHO guidelines recommend that people likely to witness an overdose, including PWID, their family and friends should have access to and training in naloxone administration to reverse the effects of an overdose [42], supporting the need for additional training for PEs.

An important finding from this study are the risks associated with PE work and the efforts by governments and NGOs to mitigate these risks. Police harassment was noted to be on the decline as drop-in-centers have actively involved police departments in awareness campaigns on harm reduction efforts within the city. As a result, and with appropriate work identification cards, PE were largely allowed to continue their outreach efforts at the drug dens. This also minimized the risk of being mistakenly arrested. However, many participants reported that interactions with police continue to be a challenge to their work, and further efforts to educate and sensitize law enforcement should be made [43]. Working with governments and police departments to ensure the safety of PEs is crucial. Understandably, drug dealers were disappointed with PEs for interfering with their clientele, creating a challenge for PEs. However, this conflict may also occur due to interpersonal issues between some PWID and drug dealers. The risk associated with accidental needlestick injuries and the possibility of contracting HIV/HCV is a significant challenge, raising the question about the welfare of PEs working with PWID. PEs in this study had access to free HIV/HCV testing and post-exposure prophylaxis (PEP) for HIV should needlestick accidents happen in the field. It is important for PE programs to incorporate training on needlestick injuries and provide access to HIV/HCV testing and PEP.

Encouraging participation of PWID in the role of PEs is associated with benefits for PEs including the emotional benefit of being productively engaged in helping PWID to access harm reduction services with some describing this work a calling. Participants reported that the guaranteed stipend at the end of the month was helpful in meeting their financial obligations and providing financial empowerment; however, many called for better remuneration and working conditions. Peer educator work is often characterized by precarious working conditions, insufficient pay and minimal job benefits [44–46]. These issues are compounded by the fact that important PE activities such as recruitment, training and retention are often not funded due to narrowly framed objectives or misalignment with research objectives [47]. Addressing these issues will be crucial to helping organizations maintain a strong network of PEs who can maintain meaningful connections between organizations and the community they serve.

Peer educators in the study highlighted the emotional burden they endured while working with PWID. They have to cope with the psychological distress resulting from the loss of clients to overdoses or infections, as well as the anguish of repeatedly working with clients relapsing or not following through with treatments. This intangible and underrecognized burden has been described by others especially within the medical field [48]. While PEs serve an important role as a source of support and counsel for PWID, this emotional challenge they face highlights the need for additional evaluation and interventions to improve PEs coping mechanisms and skills to minimize burn out. Although formal psychological counselling is rarely available to PEs, informal psychological support is often provided by other PEs or other members of staff in the DICs. However, formal counseling is needed to ensure the PEs’ mental well-being.

An important strength of this study is that we collected views from a group of male and female PEs who were formerly PWID and now working with PWID in Nairobi, Kenya, eliciting unique perspectives. Few studies have been performed among PEs working with PWID, hence our rich data add to the limited literature on the roles and risks associated with outreach work among PWID in Africa. There is limited literature on occupational roles and risks of PEs working amongst PWID. This paper may provide a framework for investigation of similar issues for PEs working with other populations.

This study also had limitations. First, our unique study setting included PEs working in Nairobi, Kenya, which may not be representative of other PE settings in Kenya or throughout East Africa; however, given that few studies have assessed the lived and professional experiences of PEs, our study may provide additional insight to improve harm reduction services. Second, we asked PEs to talk about their work and work environment. For some, this might have led to limited disclosure for fear of victimization (desirability bias). Third, due to the nature of the study design, we could not estimate the prevalence of the experiences we’ve described, or the impacts of the harm reduction program on outcomes or quality of life for PWID. Similarly, we were unable to determine whether solutions proposed by PEs were able to be implemented, or whether these solutions affected outcomes for PWID.

Despite these limitations, this study provides valuable perceptions into the daily roles and challenges faced by PEs working among PWID. Findings from this study indicate several key areas that need to be addressed. Recommendations include 1) systematically train and employ PEs to provide evidence-based patient navigation services that address the unique medical needs of PWID communities including first aid skills, identifying and initial management of drug overdoses in the field, and how to navigate healthcare settings; 2) provide training and protective gear to PEs to prevent needle stick injuries and provide access to and education about HIV/HCV testing and PEP; 3) collaborate with governments and police departments to address police harassment; 4) provide occupational mental health services to prevent drug relapse and address the emotional toll associated with losing clients. In addition, further research is warranted to better understand whether solutions proposed by PEs have been implemented and what impact, if any, they have had on outcomes for PEs and the PWID population.

In conclusion, this study found that PEs understood their roles and responsibilities, performed their duties and often times went beyond the call of duty to provide outstanding care to the clients they served, partly because they could relate to their situation.

## Supporting information

S1 Fig Flow diagram of data analyses. (TIF)

S1 Appendix In-depth interview guide questions. (DOCX)

S1 File Inclusivity survey. (DOCX)

## References

[1] UNAIDS. Global AIDS Update 2020. Available: https://www.unaids.org/sites/default/files/media_asset/2020_global-aids-report_en.pdf

[2] UNAIDS. UNAIDS Data 2019. Available: https://www.unaids.org/sites/default/files/media_asset/2019-UNAIDS-data_en.pdf

[3] Kurth AE, Cleland CM, Des Jarlais DC, Musyoki H, Lizcano JA, Chhun N, et al. HIV Prevalence, Estimated Incidence, and Risk Behaviors Among People Who Inject Drugs in Kenya. J Acquir Immune Defic Syndr. 2015;70: 420–427. doi: 10.1097/QAI.0000000000000769 26226249 PMC4624615

[4] Musyoki H, Bhattacharjee P, Sabin K, Ngoksin E, Wheeler T, Dallabetta G. A decade and beyond: learnings from HIV programming with underserved and marginalized key populations in Kenya. J Int AIDS Soc. 2021;24. doi: 10.1002/jia2.25729 34189847 PMC8242977

[5] Akiyama MJ, Cleland CM, Lizcano JA, Cherutich P, Kurth AE. Prevalence, estimated incidence, risk behaviours, and genotypic distribution of hepatitis C virus among people who inject drugs accessing harm-reduction services in Kenya: a retrospective cohort study. Lancet Infect Dis. 2019;19: 1255–1263. doi: 10.1016/S1473-3099(19)30264-6 31540840 PMC7099605

[6] Lucas GM, Cheever LW, Chaisson RE, Moore RD. Detrimental Effects of Continued Illicit Drug Use on the Treatment of HIV-1 Infection: JAIDS Journal of Acquired Immune Deficiency Syndromes. 2001;27: 251–259. doi: 10.1097/00126334-200107010-00006 11464144

[7] Lucas GM, Gebo KA, Chaisson RE, Moore RD. Longitudinal assessment of the effects of drug and alcohol abuse on HIV-1 treatment outcomes in an urban clinic: AIDS. 2002;16: 767–774. doi: 10.1097/00002030-200203290-00012 11964533

[8] Palepu A, Horton NJ, Tibbetts N, Meli S, Samet JH. Uptake and adherence to highly active antiretroviral therapy among HIV-infected people with alcohol and other substance use problems: the impact of substance abuse treatment. Addiction. 2004;99: 361–368. doi: 10.1111/j.1360-0443.2003.00670.x 14982549

[9] Mathers BM, Degenhardt L, Ali H, Wiessing L, Hickman M, Mattick RP, et al. HIV prevention, treatment, and care services for people who inject drugs: a systematic review of global, regional, and national coverage. The Lancet. 2010;375: 1014–1028. doi: 10.1016/S0140-6736(10)60232-2 20189638

[10] Ayon S, Jeneby F, Hamid F, Badhrus A, Abdulrahman T, Mburu G. Developing integrated community-based HIV prevention, harm reduction, and sexual and reproductive health services for women who inject drugs. Reprod Health. 2019;16: 59. doi: 10.1186/s12978-019-0711-z 31138238 PMC6538559

[11] Blankenship KM, Reinhard E, Sherman SG, El-Bassel N. Structural Interventions for HIV Prevention Among Women Who Use Drugs: A Global Perspective. J Acquir Immune Defic Syndr. 2015;69 Suppl 2: S140–145. doi: 10.1097/QAI.0000000000000638 25978480

[12] WHO. Guide to Starting and Managing Needle and Syringe Programmes. 2007. Available: https://apps.who.int/iris/bitstream/handle/10665/43816/9789241596275_eng.pdf;jsessionid=5CD98BAF5BE1AA5A5ADE771F5B7D9276?sequence=1

[13] Genberg BL, Shangani S, Sabatino K, Rachlis B, Wachira J, Braitstein P, et al. Improving Engagement in the HIV Care Cascade: A Systematic Review of Interventions Involving People Living with HIV/AIDS as Peers. AIDS Behav. 2016;20: 2452–2463. doi: 10.1007/s10461-016-1307-z 26837630 PMC4970970

[14] Tapera T, Willis N, Madzeke K, Napei T, Mawodzeke M, Chamoko S, et al. Effects of a Peer-Led Intervention on HIV Care Continuum Outcomes Among Contacts of Children, Adolescents, and Young Adults Living With HIV in Zimbabwe. Glob Health Sci Pract. 2019;7: 575–584. doi: 10.9745/GHSP-D-19-00210 31852741 PMC6927836

[15] Walsh N, Gibbie TM, Higgs P. The development of peer educator-based harm reduction programmes in Northern Vietnam. Drug Alcohol Rev. 2008;27: 200–203. doi: 10.1080/09595230701829348 18264883

[16] Pitpitan EV, Mittal ML, Smith LR. Perceived Need and Acceptability of a Community-Based Peer Navigator Model to Engage Key Populations in HIV Care in Tijuana, Mexico. J Int Assoc Provid AIDS Care. 2020;19: 2325958220919276. doi: 10.1177/2325958220919276 32314646 PMC7175050

[17] Chang J, Shelly S, Busz M, Stoicescu C, Iryawan AR, Madybaeva D, et al. Peer driven or driven peers?: A rapid review of peer involvement of people who use drugs in HIV and harm reduction services in low- and middle-income countries. Harm Reduct J. 2021;18. doi: 10.1186/s12954-021-00461-z 33536033 PMC7857348

[18] Jozaghi E, Greer AM, Lampkin H, Buxton JA. Activism and scientific research: 20 years of community action by the Vancouver area network of drug users. Subst Abuse Treat Prev Policy. 2018;13. doi: 10.1186/s13011-018-0158-1 29788975 PMC5964704

[19] Soukup-Baljak Y, Greer AM, Amlani A, Sampson O, Buxton JA. Drug quality assessment practices and communication of drug alerts among people who use drugs. Int J Drug Policy. 2015;26: 1251–1257. doi: 10.1016/j.drugpo.2015.06.006 26205676

[20] Bardwell G, Kerr T, Boyd J, McNeil R. Characterizing peer roles in an overdose crisis: preferences for peer workers in overdose response programs in emergency shelters. Drug Alcohol Depend. 2018;190: 6–8. doi: 10.1016/j.drugalcdep.2018.05.023 29960202 PMC6091635

[21] Kennedy MC, Boyd J, Mayer S, Collins A, Kerr T, McNeil R. PEER WORKER INVOLVEMENT IN LOW-THRESHOLD SUPERVISED CONSUMPTION FACILITIES IN THE CONTEXT OF AN OVERDOSE EPIDEMIC IN VANCOUVER, CANADA. Soc Sci Med. 2019;225: 60–68. doi: 10.1016/j.socscimed.2019.02.014 30798157 PMC6415769

[22] McLeod KE, Timler K, Korchinski M, Young P, Milkovich T, McBride C, et al. Supporting people leaving prisons during COVID-19: perspectives from peer health mentors. Int J Prison Health. 2021;ahead-of-print. doi: 10.1108/IJPH-09-2020-0069 33656310 PMC8753623

[23] Batchelder AW, Cockerham-Colas L, Peyser D, Reynoso SP, Soloway I, Litwin AH. Perceived benefits of the hepatitis C peer educators: a qualitative investigation. Harm Reduct J. 2017;14: 67. doi: 10.1186/s12954-017-0192-8 28962652 PMC5622540

[24] Mabuie M. Role of peer educators in behaviour change communication interventions for HIV prevention among people who inject drugs: Systematic review article. TSSJ. 2020;10: 189–200. doi: 10.47577/tssj.v10i1.1404

[25] Goswami P, Medhi GK, Armstrong G, Setia MS, Mathew S, Thongamba G, et al. An assessment of an HIV prevention intervention among people who inject drugs in the states of Manipur and Nagaland, India. Int J Drug Policy. 2014;25: 853–864. doi: 10.1016/j.drugpo.2014.04.016 24925819

[26] Musyoki H, Bhattacharjee P, Blanchard AK, Kioko J, Kaosa S, Anthony J, et al. Changes in HIV prevention programme outcomes among key populations in Kenya: Data from periodic surveys. PLoS ONE. 2018;13: e0203784. doi: 10.1371/journal.pone.0203784 30231072 PMC6145580

[27] National AIDS and STI Control Programme (NASCOP). Kenya National Guidelines For The Comprehensive Management of The Health Risks and Consequences of Drug Use. 2013. Available: https://www.mindbank.info/item/2441

[28] Stengel CM, Mane F, Guise A, Pouye M, Sigrist M, Rhodes T. “They accept me, because I was one of them”: formative qualitative research supporting the feasibility of peer-led outreach for people who use drugs in Dakar, Senegal. Harm Reduct J. 2018;15: 9. doi: 10.1186/s12954-018-0214-1 29486774 PMC5830063

[29] Greer AM, Amlani A, Burmeister C, Scott A, Newman C, Lampkin H, et al. Peer engagement barriers and enablers: insights from people who use drugs in British Columbia, Canada. Can J Public Health. 2019;110: 227–235. doi: 10.17269/s41997-018-0167-x 30610564 PMC6964581

[30] Austin T, Boyd J, People with Lived Expertise of Drug Use National Working Group. Having a voice and saving lives: a qualitative survey on employment impacts of people with lived experience of drug use working in harm reduction. Harm Reduct J. 2021;18: 1. doi: 10.1186/s12954-020-00453-5 33407500 PMC7789009

[31] National AIDS and STI Control Programme (NASCOP). MANUAL FOR TRAINING PEER EDUCATORS for Programs with People who Inject Drugs. 2017. Available: https://hivpreventioncoalition.unaids.org/wp-content/uploads/2019/01/NASCOP2017_Manual-for-Training-Peer-Educators-for-Programs-with-Female-Sex-Workers-Participants-Handbook_Kenya.pdf

[32] Dicicco-Bloom B, Crabtree BF. The qualitative research interview. Med Educ. 2006;40: 314–321. doi: 10.1111/j.1365-2929.2006.02418.x 16573666

[33] Guest G, Bunce A, Johnson L. How Many Interviews Are Enough?: An Experiment with Data Saturation and Variability. Field Methods. 2006;18: 59–82. doi: 10.1177/1525822X05279903

[34] Guest G, MacQueen KM, Namey EE. Applied thematic analysis. Los Angeles, Calif.; London: Sage; 2012.

[35] Golafshani N. Understanding Reliability and Validity in Qualitative Research. TQR. 2015 [cited 16 Apr 2021]. doi: 10.46743/2160-3715/2003.1870

[36] Kaiser K. Protecting respondent confidentiality in qualitative research. Qual Health Res. 2009;19: 1632–1641. doi: 10.1177/1049732309350879 19843971 PMC2805454

[37] Medley A, Kennedy C, O’Reilly K, Sweat M. Effectiveness of Peer Education Interventions for HIV Prevention in Developing Countries: A Systematic Review and Meta-Analysis. AIDS Educ Prev. 2009;21: 181–206. doi: 10.1521/aeap.2009.21.3.181 19519235 PMC3927325

[38] Ayon S, Ndimbii J, Jeneby F, Abdulrahman T, Mlewa O, Wang B, et al. Barriers and facilitators of access to HIV, harm reduction and sexual and reproductive health services by women who inject drugs: role of community-based outreach and drop-in centers. AIDS Care. 2018;30: 480–487. doi: 10.1080/09540121.2017.1394965 29067855

[39] Bartlett N, Xin D, Zhang H, Huang B. A qualitative evaluation of a peer-implemented overdose response pilot project in Gejiu, China. International Journal of Drug Policy. 2011;22: 301–305. doi: 10.1016/j.drugpo.2011.04.005 21658931

[40] Scheibe A, Shelly S, Hugo J, Mohale M, Lalla S, Renkin W, et al. Harm reduction in practice–The Community Oriented Substance Use Programme in Tshwane. African Journal of Primary Health Care & Family Medicine. 2020;12. doi: 10.4102/phcfm.v12i1.2285 32501031 PMC7284158

[41] Bouzanis K, Joshi S, Lokker C, Pavalagantharajah S, Qiu Y, Sidhu H, et al. Health programmes and services addressing the prevention and management of infectious diseases in people who inject drugs in Canada: a systematic integrative review. BMJ Open. 2021;11: e047511. doi: 10.1136/bmjopen-2020-047511 34556508 PMC8461723

[42] World Health Organization. Community management of opioid overdose. 2014. Available: https://www.who.int/publications-detail-redirect/9789241548816 25577941

[43] Cepeda JA, Strathdee SA, Arredondo J, Mittal ML, Rocha T, Morales M, et al. Assessing police officers’ attitudes and legal knowledge on behaviors that impact HIV transmission among people who inject drugs. Int J Drug Policy. 2017;50: 56–63. doi: 10.1016/j.drugpo.2017.09.009 29028564 PMC5705567

[44] George A, Blankenship KM. Peer Outreach Work as Economic Activity: Implications for HIV Prevention Interventions among Female Sex Workers. PLoS One. 2015;10. doi: 10.1371/journal.pone.0119729 25775122 PMC4361609

[45] Greer A, Bungay V, Pauly B, Buxton J. “Peer” work as precarious: A qualitative study of work conditions and experiences of people who use drugs engaged in harm reduction work. Int J Drug Policy. 2020;85: 102922. doi: 10.1016/j.drugpo.2020.102922 32911320

[46] Olding M, Barker A, McNeil R, Boyd J. Essential work, precarious labour: The need for safer and equitable harm reduction work in the era of COVID-19. Int J Drug Policy. 2020;90: 103076. doi: 10.1016/j.drugpo.2020.103076 33321286 PMC8117049

[47] Brown G, Crawford S, Perry G-E, Byrne J, Dunne J, Reeders D, et al. Achieving meaningful participation of people who use drugs and their peer organizations in a strategic research partnership. Harm Reduction Journal. 2019;16: 37. doi: 10.1186/s12954-019-0306-6 31182099 PMC6558880

[48] Tawfik DS, Scheid A, Profit J, Shanafelt T, Trockel M, Adair KC, et al. Evidence Relating Health Care Provider Burnout and Quality of Care: A Systematic Review and Meta-analysis. Ann Intern Med. 2019;171: 555–567. doi: 10.7326/M19-1152 31590181 PMC7138707

