# Supplementary figures and images for: Occupational roles and risks of community-embedded peer educators providing HIV, hepatitis C and harm reduction services to persons who inject drugs in Nairobi, Kenya
Source: PLoS One. 2022 Dec 1;17(12):e0278210. doi: 10.1371/journal.pone.0278210 (PMC9714845; doi:10.1371/journal.pone.0278210)

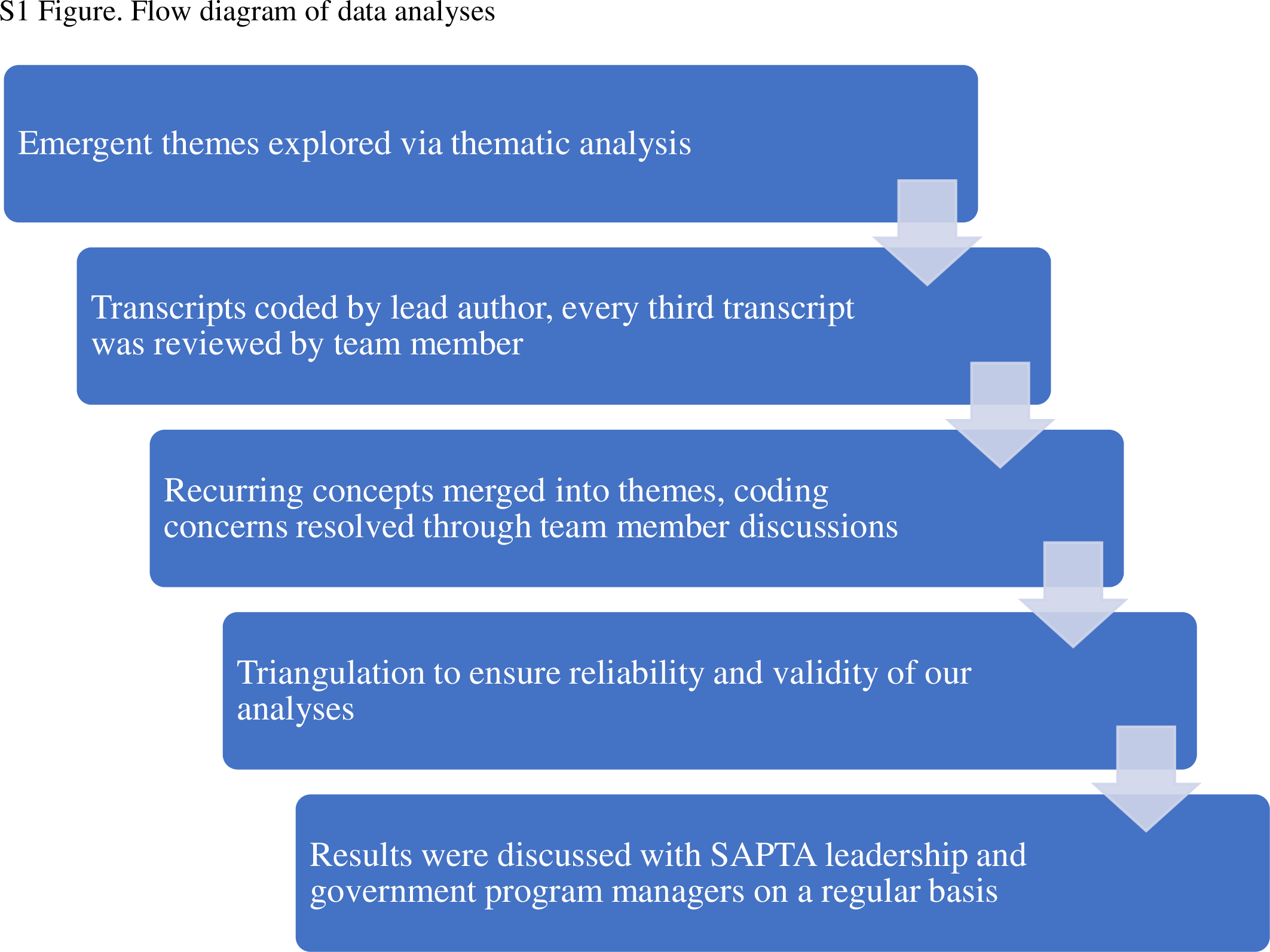

Supplement: S1 Fig — (TIF) [file pone.0278210.s001.tif]
