# Supplementary material for: Occupational roles and risks of community-embedded peer educators providing HIV, hepatitis C and harm reduction services to persons who inject drugs in Nairobi, Kenya
Source: PLoS One. 2022 Dec 1;17(12):e0278210. doi: 10.1371/journal.pone.0278210 (PMC9714845; doi:10.1371/journal.pone.0278210)
Supplement: S1 Appendix — (DOCX) [file pone.0278210.s002.docx]

**S1 Appendix: In-depth Interview Guide Questions**

*1. Please tell me a story about yourself, particularly pertaining to your use of drugs. For instance, you could tell me about:*

*a. How you first started using drugs*

*b. How you stopped using drugs*

*c. Any other story about something that happened because of your drug use.*

*2. Now I want to hear stories of people you know who have gotten sick.*

*a. Have you worked with any clients who got sick? If so, did they have to be hospitalized? What was wrong with them? Were you able to help in any way?*

*b. Have you worked with any clients who overdosed on drugs? If so, please tell me about what happened. Were you able to help them?*

*c. How frequent is overdose in this population? What is typically done about it?*

*5. Now I am going to ask you some questions about HIV.*

*a. What do you think is the main thing putting PWID at risk for HIV? Prompt: Are there behaviors you are aware of that are particularly high risk?*

*b. Are there any new drugs or drug use behaviors you’ve noticed that you’re worried about? If so, please describe and explain why this worries you.*

*b. What do you think would be the best way for an organization like SAPTA to help PWID protect themselves against HIV?*

*6. Now I am going to ask you some questions about the clients who you work with through SAPTA who are injecting drugs.*

*a. What do you think are the biggest problems facing PWID currently?*

*b. Is it easy or difficult for PWID to access services to help them, such as NSP and HIV testing? Why is it easy/difficult?*

*c. What do you think would help clients the most?*

*d. Are there things SAPTA should be doing to help clients that aren’t currently offered? If so, what are they?*

*7. Now I’m going to ask you about your work as a Peer Educator.*

*c. What are the main challenges in your daily work as a PE?*

*d. What would make your job as a PE easier?*

*e. Do you have other employment, or is this your only source of income?*

*f. Have you ever considered leaving SAPTA to work somewhere else?*

*g. IF SO: Why did you consider that?*

*8. Is there anything else that you think it’s important for researchers to know?*
