# Supplementary material for: Occupational roles and risks of community-embedded peer educators providing HIV, hepatitis C and harm reduction services to persons who inject drugs in Nairobi, Kenya
Source: PLoS One. 2022 Dec 1;17(12):e0278210. doi: 10.1371/journal.pone.0278210 (PMC9714845; doi:10.1371/journal.pone.0278210)
Supplement: S1 File — (DOCX) [file pone.0278210.s003.docx]

Inclusivity in global research

PLOS’ policy on inclusivity in global research aims to improve transparency in the reporting of research performed outside of researchers’ own country or community and ensures that PLOS publications reporting global research adhere to high standards for research ethics and authorship. Authors of relevant research articles may be asked to complete the questionnaire below, which outlines ethical, cultural, and scientific considerations specific to inclusivity in global research. This questionnaire may be requested when researchers have travelled to a different country to conduct research, if research uses samples collected in another country, research with Indigenous populations or their lands, or if research is on cultural artefacts. Researchers travelling to another country solely to use laboratory equipment will not normally be required to complete the questionnaire. However, the questionnaire can be requested at the journal’s discretion for any submission – if you have been requested to complete this questionnaire by the PLOS journal you submitted to, please do so.

Please complete the questionnaire below and include this as a Supporting Information file with your manuscript. Note that if your paper is accepted for publication, this checklist will be published with your article in the supporting information files. Please ensure that you reference the checklist in the main body of your manuscript. We suggest adding a subsection ‘Inclusivity in global research’ to your Methods section and adding the following sentence: “Additional information regarding the ethical, cultural, and scientific considerations specific to inclusivity in global research is included in the Supporting Information (SX Checklist)”

The questions have been designed to be applicable to a wide range of study types, and there are subsections for both human subjects research and non-human subjects research. If any of the questions are not relevant to your research please mark them as “N/A” as appropriate.

**Ethical considerations, permits and authorship**

*This section is applicable to all research types.*

Provide details as to who granted permissions and/or consent for the study to take place in the Methods section of your manuscript. This should include the names of **all** ethics boards, governmental organizations, community leaders or other bodies that provided approval for the study. If individuals provided approval refer to these people by their role or title but do not list their name(s).

Reported on page number: 7

If there were any deviations from the study protocol after approval was obtained please provide details of these changes in the Methods section of your manuscript.
Did this study involve local collaborators that are residents of the country where the research was conducted or members of the community studied? If you do not have any authors from said communities, please provide an explanation for this below.

Reported on page number: Not applicable

Our research was conducted with a binational study team based in the US and Kenya. The first author (LNM), coordinator (LM), community partners (WS, EG), consultant (DB) and Ministry of Health representative (HM) are all from Kenya, with extensive experience working with PWID. All other collaborators have lived and worked in Kenya (AMW has lived and worked in Kenya for >10 years, CF worked in Kenya for >20 years, BG lived and worked in Kenya for >10 years, and NLB worked in Kenya for >4 years.)

Everyone listed as an author should meet PLOS’ criteria for authorship and all individuals who meet these criteria should be included in the author byline, rather than the acknowledgements. Authorship criteria is based on the International Committee of Medical Journal Editors (ICMJE) Uniform Requirements for Manuscripts Submitted to Biomedical Journals - for further information please see here: <https://journals.plos.org/plosone/s/authorship>.

**Human subjects research (e.g. health research, medical research, cross-cultural psychology)**

Did you obtain written informed consent from a representative of the local community or region before the research took place? How did you establish who speaks for the community? Details of written informed consent obtained from study participants should be reported separately in the Methods section of your manuscript.

All study procedures and materials, including consent forms, were approved by the University of Washington Institutional Review Board (Seattle, WA, USA) and the Kenyatta National Hospital Ethical Review Committee (Nairobi, Kenya). In addition, all study materials and procedures were drafted with community partners and the oversight of government (MOH) personnel and each participant provide written informed consent in Enligsh or Swahili.

How did members of the local community provide input on the aims of the research investigation, its methodology, and its anticipated outcome(s)?

This work was in collaboration with local government (i.e., Kenya Ministry of Health, NASCOP), established community partners (SAPTA), Kenyatta National Hopsital (Nairobi, Kenya), and the University of Washington (Seattle, WA, USA). Study inception, development of aims, protocols, study materials and the interpretation of findings were all conducted as an interative and collaborative process, with each entity contributing their expertise. Findings have been presented at domestic and international conferences, with the support of Kenya’s MOH and SAPTA.

When engaging with the local community, how did you ensure that the informed consent documents and other materials could be understood by local stakeholders?

Study matierals were edited by all collaborators including government officials, community partners and local consultants. This work is a secondary analysis, with the primary *a priori* analysis centered around barriers and facilitators to HIV and hepatitis C care. Together, all research collaborators were convinced that the emergent topic of occupational risk among PEs needed to be discussed on a global platform, to disseminate key findings.

Will the findings of the research be made available in an understandable format to stakeholders in the community where the study was conducted (e.g. via a presentation, summary report, copies of publications, etc.)? Please provide details of how this will be achieved.

This work has been shared at two local confereneces in Kenya, which were attended by local medical community members who provice services to PWID. In addition, much of the findings were converted into low literacy pamphlets with picture graphics to share with PWID communities. In addition, this manuscript will help our community partners advocate for additional support for PE programs and services.

**Non-human subjects research using specimens/ animals collected as part of the study, or those housed in archival collections. Examples include archaeology, paleontology, botany and zoology.**

Did the permission you obtained from a local authority to perform the study include an agreement on access to outputs and benefit sharing? This may include procedures to enable fair distribution of the benefits and resources arising from the research performed. Please include any details of Prior Informed Consent and Benefit Sharing Agreements obtained. These may be required by field-specific regulations, for example the Convention on Biological Diversity (CBD) and the associated Nagoya Protocol.

Not applicable.

If the material used in your study was imported, please A) provide the year it was imported and B) indicate whether permits were obtained to import/export the materials used, C) provide details of any permits obtained. If this information is not available, please indicate this.

Not applicable.

If you used archival specimens, please state how the material used in your study was acquired by the institute it is held in and provide details of any permits obtained for the original excavations/ sample collection. If this information is not available, please indicate this.

Not applicable.

How was the potential cultural significance of the materials collected in your study to local communities considered in your research design? Were Indigenous peoples and/or local researchers and institutions involved with archaeological excavations / collection of specimens? If so, please provide a description of their involvement.

We involved our community partners and informed govenerment officials in each step of the research study design, including the development of study aims, protocols, questionnaires, findings, etc. Our community partners allowed us to conduct research within their facilities, and communication was constant about study recruitment and enrollment. Government officials were informed of study progress during monthly working group meetings. Consultants were also informed of study progress on a monthly basis.

If your manuscript includes photographs of human remains please indicate whether authors obtained permission from descendants or affiliated cultural communities to do so.

Not applicable.
